# Letter to Editor on the “Prevalence and Associated Risk Factors of Suicidal Tendencies Among Bangladeshi Public University Students: A Mixed‐Methods Exploration”

**DOI:** 10.1002/hsr2.72600

**Published:** 2026-06-02

**Authors:** Rinoa Vun Hui Jun, Agong bin Lupat, Deeni Rudita bin Idris

**Affiliations:** ^1^ Faculty of Nursing, PAPRSB Institute of Health Science, Universiti Brunei Darussalam Bandar Seri Begawan Brunei Darussalam

To the Editor,

This letter to the editor is in reference to an article titled “**Prevalence and Associated Risk Factors of Suicidal Tendencies Among Bangladeshi Public University Students: A Mixed‐Methods Exploration**” which I encountered in your esteemed journal [1]. This study examines the prevalence and associated risk factors of suicidal tendencies among public university students in Bangladesh.

There are two key points that I would like to humbly highlight, and if clarified, would strengthen the paper's contributions. The first point involves the contradictory *p*‐value statements for *χ* ^2^ = 47.718, and the second involves the inconsistencies in how ‘*Duration*' was collected and labelled.

In the *Results*, specifically under the Association Between Suicidal Agents, Suicidal Prevalence, Age and Duration of Using Online Platforms section, the authors of this article mentioned “The chi‐square statistic Q31*age was significant (47.718, *p* < 0.004), indicating that this interaction had a significant effect on the agent for changing suicidal patterns.” [1], p. 8. However, this contradicts the *Discussion* section that states that “Finally, age had a significant association with suicidal prevalence level (*χ* ^2^ = 47.718, df = 25, *p* > 0.004)” [1], p. 12. Both sentences reported the same χ^2^ value, but the inequality signs for the p‐value differ (*p* < 0.004 vs *p* > 0.004). Furthermore, the consensus is that “If *p* < 0.05, then it signifies association and if *p* > 0.05, then it signifies no association.” [1], *p*. 11. Which means that *p* > 0.004 would not be considered as ‘significant'. Thus, I would like to kindly seek clarification on the varying symbols in the *p*‐value, as it may create confusion for the readers on whether the interaction was significant or not.

In addition, page 6 of the *Results* section showcases the demographic information of the participants in this study. One of the demographic variables includes the Q18: ‘Duration of using Online Platforms' that was categorised (**Less than 1 h**, **1–2 h**, **3–4 h**, **5–6 h**, and **More than 7 h**) and given Frequency (*n*) and Percentage (%) values [1]. As shown in Table 4 of the original article, Q18 was considered as a continuous covariate, and the authors state that “As duration increased by 2.333 units, the odds ratio/probability to select economic factors compared to technological, cultural, social agency, and relationship problems was more by 2.33 times.” [1], p. 8. Initially, the duration variable was considered a categorical variable, as demonstrated in Table 2; however, it was then labelled as a continuous variable afterwards, as depicted in Table 4 [1]. I would like to gently highlight that this discrepancy was not clarified anywhere on how the ‘Duration' categories were converted/transformed into numbers. This may possibly cause the odds ratio of interpretation (OR = 2.333; 95% CI = 1.109–4.908, *p* = 0.026) to be unclear. Therefore, I would like to humbly request clarification on how Q18 was coded into numbers for regression.

Given the relevance of this study, I would also like to politely suggest that the authors examine the help‐seeking behaviours (e.g., via the General Help‐Seeking Questionnaire) of these students alongside online exposure to cyberbullying and suicidal tendencies for future studies. Currently, many studies and universities are increasingly prioritising the help‐seeking tendencies of students in Higher Education Institutions (HEIs) when faced with psychological distress or mental health concerns [2, 3]. By associating these two factors with HEI students' help‐seeking preferences, this may help higher educational institutions determine how the study outcomes influence university students' help‐seeking intentions. This is corroborated by Rastogi et al. [4], where tertiary students with suicidal behaviours or thoughts associate with low help‐seeking behaviours. It could also push this research towards an outcome that informs mental health changes among tertiary institutions and their respective counselling services. For instance, Novak and Kovács [5] advocate for universities and their counselling services to adapt to their students' cultural, academic, and mental health needs through visibility, accessibility, and linguistic inclusivity.

In conclusion, I would like to respectfully seek clarification regarding the discrepancies in the significance of the *p*‐values and how the ‘Duration of using Online Platforms' in Q18 was converted from categorical variables to Covariates (continuous). I have also humbly provided a short suggestion that the study's outcomes may highlight the need for exploring the help‐seeking behaviours of university students.

## Author Contributions

**Rinoa Vun Hui Jun:** conceptualisation, writing – original draft, writing – review and editing; **Agong bin Lupat:** supervision; **Deeni Rudita bin Idris:** supervision.

## Funding

The authors have nothing to report.

## Ethics Statement

Ethical approval was not required for this letter as it does not contain original human or animal research data.

## Conflicts of Interest

The authors declare no conflicts of interest.

## Authors Approved Statement

All authors have read and approved the final version of the manuscript. Rinoa Vun Hui Jun had full access to all of the data in this study and takes complete responsibility for the integrity of the data and the accuracy of the data analysis.

## Transparency Statement

The lead author (**Rinoa Vun Hui Jun**) affirms that this manuscript is an honest, accurate, and transparent account of the study being reported; that no important aspects of the study have been omitted; and that any discrepancies from the study as planned (and, if relevant, registered) have been explained.

## Data Availability

Data sharing not applicable to this article as no datasets were generated or analysed during the current study.
